# Atypical neurological manifestations in anti-IgLON5 disease: a case report

**DOI:** 10.3389/fneur.2024.1340284

**Published:** 2024-02-21

**Authors:** Yun Chen, Jingyao Chen, Zhaohua Pei, Wei Qian

**Affiliations:** ^1^Department of General Practice, The First People’s Hospital of Hangzhou Lin’an District, Hangzhou Medical College, Hangzhou, Zhejiang, China; ^2^Department of Emergency Medicine, The First People’s Hospital of Hangzhou Lin’an District, Hangzhou Medical College, Hangzhou, Zhejiang, China

**Keywords:** autoimmune encephalitis, anti-IgLON5 disease, IgLON5, cognitive impairment, case report

## Abstract

Anti-IgLON5 disease is a recently discovered autoimmune encephalopathy with sleep disorder as a hallmark in the majority of reported cases. Additional neurological manifestations include bulbar dysfunction, gait problems, movement disorders, oculomotor abnormalities, and hyperexcitability of the nervous system. At present, an increasing number of publications have dealt with the course and possible treatment options for anti-IgLON5 disease, and its clinical spectrum has expanded wider and more heterogeneous. Here, we report a case of a 66-year-old female with cognitive impairment accompanied by slow reaction, impaired memory, and decreased orientation. A positive cerebral MRI change and serum and cerebrospinal fluid (CSF) antibodies against IgLON5 were found during the diagnostic course. Subsequently the patient received immunotherapy and was generally in good health with no new symptoms during follow-up. Early testing for IgLON5 antibodies should be considered in patients with atypical neurological symptoms such as cognitive impairment, slow reaction, or decreased orientation. In clinical practice, immunotherapy should be considered in all cases of anti-IgLON5 encephalopathies.

## Introduction

Anti-IgLON5 disease is a novel condition presenting with prominent sleep disturbances and gait problems, along with anti-IgLON5 antibodies and pathological features of neuronal tauopathy. Since first reported by Lidia Sabater in 2014 (1), an increasing number of anti-IgLON5 disease cases have been reported, thereby expanding its clinical spectrum (2–5) . Cases of anti-IgLON5 disease have been reported in America, Asia, the UK, and Europe, with a median age between 45 and 83 years at diagnosis (6). The prevalence of anti-IgLON5 antibodies is estimated to be 12 out of every 150,000 specimens per year (7). Due to numerous cases being misdiagnosed and misreported, it is estimated that the true prevalence of Ig-LON5 disease may be higher.

This is a novel autoimmune disorder characterized by a complex interplay between inflammation and neurodegeneration (8). Furthermore, the disease onset tends to be more protracted rather than acute. Although the anti-IgLON5 antibody is classified as a cell surface antibody, the clinical syndrome does not exhibit encephalitis, such as lymphocytic inflammatory infiltration. The observation that IgLON5 antibody effects were irreversible. Immunotherapy could potentially stabilize or reduce the IgLON5 levels, thereby reducing auto-antibodies-induced neuron damage, and preventing irreversible neurology sequela (9). Given the limited experience in treating anti-IgLON5 disease, it is advisable to promptly commence immunotherapy as the first line of action as soon as possible.

Herein, we present a case of anti-IgLON5 disease manifested by cognitive impairment accompanied by slow reaction, impaired memory, and decreased orientation. Testing for IgLON5 antibodies should be considered in patients with atypical neurological symptoms such as cognitive decline, and medical intervention is necessary to achieve a favorable prognosis.

## Patient information

A 66-year-old female was admitted to the First People’s Hospital of Hangzhou Lin’an District in March 2023 with complaints of cognitive impairment for 4 days. The patient had no concurrent symptoms, such as fever, headache, sleep disorder, mental disorder, tinnitus, visual rotation, and autonomic dysfunction. She works as a farmer. She had a documented history of effectively managing hypertension for over a decade with no other chronic diseases or tumors. There was no family history of similar complaints, seizures, early-onset dementia, and other neurological disorders.

## Timeline

**Figure 1:**
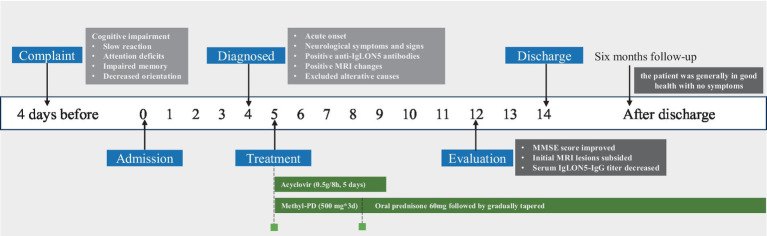
Timeline displaying the evolution of this case: in the white boxes, the date has been reported; in the blue boxes is a list of the important events that have been performed; in the green boxes, we have displayed the therapies; and in the black boxes, the evaluation was conducted, MRI, magnetic resonance imaging; Methyl-PD, methylprednisolone; MMSE, Mini-mental stare examination.

The timeline of this case report was shown in Figure 1.

## Diagnostic assessment

**Figure 2:**
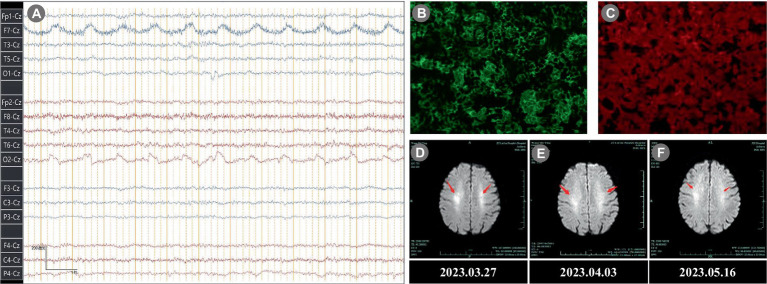
Electroencephalogram, Immunofluorescence, and MRI results. **(A)** Electroencephalogram revealed a fundamental rhythm with a small amount of low-amplitude α (7–14 Hz) and θ activity (4–7 Hz). **(B)** Immunofluorescence of Serum anti-IgLON5 antibody (1:1,000). **(C)** Immunofluorescence of CSF anti-IgLON5 antibody (1:100). **(D–F)** Brain MRI-DWI showed symmetrical high signal intensity in the both sides of the centrum semiovale (arrowhead). **(D)** 2023.03.27; **(E)** 2023.04.03; **(F)** 2023.05.16. MRI, magnetic resonance imaging; DWI, diffusion-weighted; CSF, cerebrospinal fluid.

In the neurological physical examination, the patient showed cognitive impairment, including slow reaction, attention deficits, impaired memory and decreased orientation. Examination of gait revealed mild instability. No abnormalities were observed in his cranial nerves, systemic sensory system, deep and shallow reflexes, muscle strength, pyramidal tract reflex and autonomic nervous function. Mini-mental state examination (MMSE) score was 12 points and the Montreal cognitive assessment (MoCA) score was 15 points. Laboratory examinations revealed an erythrocyte sedimentation rate (ESR) level of 48 mm/h (<38 mm/h), a creatine kinase level of 3,310 U/L (40–200 U/L), a lactate dehydrogenase (LDH) level of 374 U/L (120–250 U/L) and a ferritin level of 418.8 ng/mL (13–150 ng/mL). The serum IgG antibody of Herpes simplex virus was 39.3 COI (≥1.10 COI) and cytomegalovirus was 94 U/mL (≥14 U/mL). The other laboratory tests were all normal or negative, including blood routine test, urine routine test, coagulation function test, D-dimer determination, vitamin B1 and B12, folic acid, antithyroid peroxidase, antinuclear antibodies (ANA), antineutrophil cytoplasmic antibodies (ANCA), and complement. All tumor markers were all seronegative. Cerebral diffusion-weighted magnetic resonance imaging (MRI-DWI) revealed hyperintensities in both sides of the centrum semiovale (Figures 2D–F). Electroencephalogram revealed a fundamental β rhythm with a small amount of low-amplitude α (7–14 Hz) and θ activity (4–7 Hz; Figure 2A). A polysomnographic (PSG) examination showed normal sleep architecture. At the moment, the diagnosis was unclear and then a lumbar puncture was performed. The intracranial pressure was measured within the normal range (120 mmH2O), and the pathogen culture of cerebrospinal fluid (CSF) was negative. The CSF results showed a slightly increased protein level of 592.22 mg/L and IgG antibody level of 61.9 mg/L. An assay panel (Hangzhou DIAN Medical Laboratory Test) of serologic and CSF tests for anti-IgLON5 IgG was positive (serum: 1:1,000, CSF: 1:100; Figures 2B,C), while other autoantibodies (Ri, Yo, Hu, malforming-A1, malforming-A2, N-methyl-D-aspartate receptor, α-amino-3—hydroxy-5-methyl-4-isoxazole propionic acid receptor, contactin-associated protein receptor 2, leucine rich glioma-inactivated protein 1, γ-amino butyric acid type B receptor, dipeptidyl-peptidase-like protein 6, glyoxylate reductase 1, metabotropic glutamate receptor 5) was negative. For economic reasons, our patient refused the genetic test, a very helpful evaluation for anti-IgLON5 disease. According to the latest consensus on AE (10), the patient met two main diagnostic indexes and reasonably ruled out alternative causes such as infectious diseases, toxicopathy, cerebral apoplexy, and tumors. A final diagnosis of anti-IgLON5 disease was established.

### Therapeutic intervention

After diagnosis, the patient immediately received a pulse dose of intravenous methylprednisolone of 500 mg/day for a duration of 3 days followed by an oral prednisolone acetate dose of 60 mg/day (tapered slowly every 2 weeks). In addition, she received empirical treatment with acyclovir of 0.5 g/8 h for 5 days.

### Follow-up and outcomes

After the above treatment, the cognitive function of our patient rapidly improved with MMSE score of 17, the serum IgLON5-IgG titer decreased to 1:10, and the initial MRI lesions subsided. At present, 6 months after discharge, the patient is generally in good health with no new symptoms.

## Discussion

Here, we report a case of anti-IgLON5 disease manifested by cognitive impairment, slow reaction, impaired memory, and decreased orientation. To our knowledge, the clinical presentation of anti-IgLON5 disease exhibits significant heterogeneity and varies widely between individuals (11). Sleep disorders are the primary and most prominent characteristic (12). A retrospective clinical analysis of 22 patients with anti-IgLON5 disease demonstrated that symptoms at initial consultation included sleep problems (36%), gait abnormality (36%), bulbar dysfunction (14%), chorea (9%), and cognitive decline (5%), but eventually developed parasomnia, excessive daytime sleepiness, sleep apnea, or insomnia (13).

Evidence revealed that anti-IgLON5 disease can be readily suspected if one presents with a distinctive sleep disorder in association with one or more of the following symptoms: gait instability, bulbar dysfunction, movement disorder, oculomotor abnormality, and cognitive dysfunction (14). At present, there are no definite diagnostic criteria for this disease, and the detection of anti-IgLON5 antibodies in CSF and serum remains a relatively recognized diagnostic foundation (15). Our patient presented cognitive impairment upon admission to the hospital, which was a relatively unspecific manifestation of the anti-IgLON5 disease and poses rapid diagnosis more challenging. These symptoms may precede or surpass the development of a sleep disorder and therefore lead to the initial visit to the doctor (13). It is essential to test for IgLON5-antibodies in patients with atypical neurological symptoms. We reason that early diagnosis is essential not only to avoid unnecessary tests, but also to prevent complications.

It was discovered that IgLON5 is mainly expressed in the nervous system during the sequencing of human chromosome 19. Although IgLON5’s exact role is unknown, previous studies suggest it could be involved in tau physiology (16). Neuropathological investigations have shown that hyperphosphorylated tau has a propensity to accumulate in structures located in the basal brain structures, such as the hypothalamus, the tegmentum and the periaqueductal gray matter (17). It remains unclear whether IgLON5 antibodies contribute to the pathogenesis of this tauopathy, however, their association with HLA-DRB1*10:01 and HLA-DQB1*05:01 strongly suggests a highly probable involvement (18). Alternatively, the development of IgLON5 antibodies is believed to induce neurodegeneration in particular regions of the central nervous system, predominantly affecting the tegmentum of the brainstem, hypothalamus, and hippocampus (19). However, the precise nature of the anti-IgLON5 disease remains elusive, as it is yet to be determined whether it manifests as a degenerative disorder with a secondary inflammatory response to IgLON5, or if it presents a primary autoimmune condition (20, 21).

Prompt initiation of immunotherapy could potentially stabilize or reduce the IgLON5 levels, thereby mitigating auto-antibodies-induced neuron damage (22). Despite the ongoing debate regarding the effectiveness of immunotherapy due to the lack of alternative treatment options, it appears to be the primary choice for treating for treating anti-IgLON5 disease. Sudden death, despite initial responses to immune treatment, has also been reported previously, however, those who had not received immunotherapies fared worse. The most commonly employed treatment methods include cycles of corticosteroid, immunoglobulins administration, therapeutic plasma exchange, cyclophosphamide, azathioprine, mycophenolate mofetil, and rituximab (7). Therapeutic combinations and second-line interventions appear to be more efficacious at providing sustained responses than monotherapy. In one of the largest systematic reviews, 43 % of patients demonstrated a responded to immunotherapy, and 15 patients had a positive response at final follow-up (23). Among the 22 patients characterized by Gaig et al., 20 patients received immunotherapy, while only two patients showed mild and transient improvement in their symptoms (13). In a retrospective cohort involving 53 anti-IgLON5 disease patients, 70% (36/53) of the patients were treated with immunotherapy. Total anti-IgLON5 IgG levels of the patients under immunotherapy were reduced, while in patients not experienced immunotherapy doubled over time. Meanwhile, an early onset of immunotherapy was a significant treatment response predictor of beneficial effects (16). In another case of anti-IgLON5 disease, the serum IgLON5-IgG titer decreased and initial MRI changes lessened following administration of methylprednisolone and immunoglobulins (24). In similar cases of anti-IgLON5 disease who presenting with cognitive dysfunction, patients who response to immunotherapy have great outcomes (25, 26).

Considering the favorable outcome of our case, it is suggested that immunotherapy should be considered for all cases of anti-IgLON5 encephalopathies in clinical practice. Notably, current evidence for predicting patients’ response to immunotherapy are mostly based on relatively small sample sizes. A large-scale cohort study is required to investigate the factors predicting the efficacy of immunotherapy for the anti-IgLON5 disease. The major limitation of the study presented here is the short follow-up, which could not comprehensively assess the patient’s long-term prognosis. Meanwhile, neuropathology or genetic testing was not performed during hospitalization. In conclusion, we report a case of anti-IgLON5 disease characterized by cognitive impairment, slow reaction, impaired memory, and decreased orientation. Understanding atypical neurologic manifestations is essential for prompt diagnosis, as these symptoms may precede or surpass the development of the sleep disorder. Detection of IgLON5-antibodies should be considered in patients with atypical neurological symptoms such as cognitive decline. In clinical practice, immunotherapy should be considered in all cases of anti-IgLON5 encephalopathies.

## Data availability statement

The raw data supporting the conclusions of this article will be made available by the authors, without undue reservation.

## Ethics statement

The studies involving humans were approved by Ethics Committee/The First People’s Hospital of Hangzhou Lin’an District, Hangzhou Medical college. The studies were conducted in accordance with the local legislation and institutional requirements. The participants provided their written informed consent to participate in this study. Written informed consent was obtained from the individual(s) for the publication of any potentially identifiable images or data included in this article.

## Author contributions

YC: Conceptualization, Data curation, Formal Analysis, Investigation, Methodology, Project administration, Resources, Software, Supervision, Validation, Visualization, Writing – original draft, Writing – review & editing. JC: Conceptualization, Methodology, Project administration, Data curation, Formal Analysis, Writing – original draft. ZP: Conceptualization, Data curation, Formal Analysis, Methodology, Writing – original draft, Investigation, Software, Supervision, Validation, Visualization. WQ: Conceptualization, Investigation, Methodology, Supervision, Validation, Visualization, Project administration, Writing – review & editing.
