# Supplementary material for: Atypical neurological manifestations in anti-IgLON5 disease: a case report
Source: Front Neurol. 2024 Feb 21;15:1340284. doi: 10.3389/fneur.2024.1340284 (PMC10915776; doi:10.3389/fneur.2024.1340284)
Supplement: Supplementary file 1 [file Table_1.docx]

Supplemental Table 1. Baseline characteristics of the patient.

| Variables | Results |
| --- | --- |
| Age (year) | 66 |
| gender | F |
| Hemoglobin (g/L) | 141 |
| CRP (mg/L) | 54.4 |
| ESR (mm/h) | <38 |
| PCT (ng/ml) | 0.05 |
| Albumin (g/L) | 39.3 |
| Creatinine (µmol/L) | 47 |
| Uric acid (µmol/L) | 315 |
| Total cholesterol (mmol/L) | 4.28 |
| Triglyceride (mmol/L) | 0.91 |
| LDH (U/L) | 374 |
| CK (U/L) | 3310 |
| AFP (ng/ml) | <1.3 |
| CEA (ng/ml) | 0.82 |
| CA19-9 (U/ml) | 12.9 |
| CA125 (U/ml) | 23.7 |
| CA15-3 (U/ml) | 8.3 |
| Herpes simplex virus IgG antibody (COI) | 39.3 |
| Herpes simplex virus IgM antibody (COI) | 0.02 |
| Cytomegalovirus IgG antibody (U/ml) | 94 |
| Cytomegalovirus IgM antibody (U/ml) | 1.12 |
| EB virus IgG antibody (U/ml) | 342 |
| EB virus IgM antibody (U/ml) | 0.65 |
| Cryptococcal antigen | N |
| Acid-fast bacilli | N |
| HBV | N |
| HCV | N |
| ANA | N |
| ANCA | N |
| Serum anti-IgLON5 antibody | 1:1000 |
| CSF |  |
| Intracranial pressure (mmH20) | 120 |
| RBC count (/L) | 0 |
| Protein (mg/L) | 599.22 |
| Glucose (mmol/L) | 5.6 |
| ADA (U/L) | 0.3 |
| LDH (U/L) | 24 |
| IgG antibody (mg/L) | 61.9 |
| Anti-IgLON5 antibody | 1:100 |

Abbreviations: F, female; CRP, C-reactive protein; ESR, erythrocyte sedimentation rate; PCT, Procalcitonin; LDH, lactate dehydrogenase; CK, creatine kinase; AFP, alpha-fetoprotein; CEA, carcinoembryonic antigen; CA, glucoprotein antigen; Ig, immunoglobulin; N, negative; EB, Epstein-barr; HBV, hepatitis B virus; HCV, hepatitis C virus; ANA, antinuclear antibody; ANVA, antineutrophil cytoplasmic antibody; CSF, cerebrospinal fluid; RBC, red blood cell; ADA, adenosine deaminase.
